# Characterising inflammatory markers in two childhood autoimmune diseases (JIA and JDM) pre and post methotrexate

**DOI:** 10.1186/1546-0096-9-S1-O8

**Published:** 2011-09-14

**Authors:** S Ursu, H Moncrieffe, H Varsani, RHB Hamaoui, L Beard, L Kassoumeri, F Patrick, LR Wedderburn

**Affiliations:** 1 Rheumatology Unit, UCL Institute of Child Health, London, United Kingdom

## Background

Methotrexate (MTX) is the first line of therapy for patients with juvenile idiopathic arthritis (JIA) and juvenile dermatomyositis (JDM). The mechanisms of action of MTX in childhood autoimmunity are not fully elucidated. The primary target sites of inflammation of JIA and JDM i.e. joint and muscle, respectively do not represent sites from which samples can be easily obtained to monitor inflammatory markers. In contrast peripheral blood is accessible and can be obtained at time of routine clinical screen. We hypothesized that changes in peripheral blood of patients would be informative in monitoring disease and understanding inflammatory pathways that are modulated by MTX.

## Aim

To characterise the inflammatory immunological profile at protein and cellular level in peripheral blood of patients with JIA and JDM pre and post MTX.

## Methods

Blood samples and clinical data were obtained from 109 JIA patients from the Sparks CHARM study and 27 JDM patients from the UK JDM Cohort study pre and at 6 months of therapy and 6 healthy control children. The study had full ethical approval and consent. Nine serum cytokines including TNF-alpha and IL-1beta were quantified by in-house Luminex (de Jager, 2007). PBMC were extracted by standard density centrifugation and intracellular cytokines measured by flow cytometry.

## Results

We observed downregulation in serum IL-6 in the 61 JIA patients with paired samples (p=0.02) pre to post MTX (14pg/ml to 10pg/ml) and a downward trend was also measured in JDM serum (p=0.1). Interestingly, IL1-beta concentrations were low in JIA (<1pg/ml) but elevated in JDM (50pg/ml pre and 61pg/ml post MTX). We measured an elevated proportion of IL-17+ CD4 T cells in JDM PBMC before therapy compared to healthy children controls (p=0.08) that did not go back to control levels even after MTX treatment.

## Conclusion

The proinflammatory cytokine blood signature appears different in JIA and JDM patients pre-MTX therapy. For JDM patients, the observation that higher levels in the blood of Th17 cells which fail to reduce after treatment, may represent a need for novel therapies which will target the Th17 pathway.

The CHARM study was supported by grants from SPARKS UK, The Big Lottery Fund, Arthritis Research UK and Great Ormond Street Hospital Children’s Charity. The UK JDM Cohort Study was supported by the Wellcome Trust, Action Medical UK and the Henry Smith Charity. We thank patients, parents and staff at Great Ormond Street Hospital NHS Trust, London and the members and co investigators of the JDM Research group.

